# Linear echoendoscope-guided ERCP for the diagnosis of occult common bile duct stones

**DOI:** 10.1186/1471-230X-13-44

**Published:** 2013-03-05

**Authors:** Hoi-Hung Chan, E-Ming Wang, Meng-Shun Sun, Ping-I Hsu, Wei-Lun Tsai, Tzung-Jiun Tsai, Kai-Ming Wang, Wen-Chi Chen, Huay-Min Wang, Huei-Lung Liang, Kwok-Hung Lai, William Robert Brugge

**Affiliations:** 1 Division of Gastroenterology, Department of Internal Medicine, Kaohsiung Veterans General Hospital, 386 Ta-Chung 1st Road, Kaohsiung 81362, Taiwan; 2 Department of Biological Sciences, National Sun Yat-sen University, 70 Lien-Hai Road, Kaohsiung, 80424, Taiwan; 3 College of Pharmacy and & Health Care, Tajen University, 20 Weisin Road, Sin-er Village, Yanpu Township, Pingtung County, 907, Taiwan; 4 School of Medicine, National Yang-Ming University, No. 155, Sec. 2, Li-Nong Street, Taipei, Pei-Tou, 112, Taiwan; 5 Department of Gastroenterology and Hepatology, Yuan’s General Hospital, 162 Cheng Kung 1st Road, Kaohsiung, 80249, Taiwan; 6 Department of Radiology, Kaohsiung Veterans General Hospital, 386 Ta-Chung 1st Road, Kaohsiung, 81362, Taiwan; 7 Division of Gastroenterology, Massachusetts General Hospital, 55 Fruit Street, Boston, MA 02114, USA

**Keywords:** Linear echoendoscope, Occult common bile duct stones

## Abstract

**Background:**

Less than 67% of patients with intermediate risk for common bile duct (CBD) stones require therapeutic intervention. It is important to have an accurate, safe, and reliable method for the definitive diagnosis of CBD stones before initiating therapeutic endoscopic retrograde cholangiopancreatography (ERCP). Few publications detail the diagnostic efficacy of linear echoendoscopy (EUS) for CBD stones.

**Methods:**

30 patients with biliary colic, pancreatitis, unexplained derangement of liver function tests, and/or dilated CBD without an identifiable cause were enrolled in the study. When a CBD stone was disclosed by linear EUS, ERCP with stone extraction was performed. Patients who failed ERCP were referred for surgical intervention. If no stone was found by EUS, ERCP would not be performed and patients were followed-up for possible biliary symptoms for up to three months.

**Results:**

The major reason for enrollment was acute pancreatitis. The mean predicted risk for CBD stones was 47% (28–61). Of the 12 patients who were positive for CBD stones by EUS, nine had successful ERCP, one failed ERCP (later treated successfully by surgical intervention) and two were false-positive cases. No procedure-related adverse events were noted. For those 18 patients without evidence of CBD stones by EUS, no false-negative case was noted during the three-month follow-up period. Linear EUS had sensitivity, specificity, positive and negative predicted values for the detection of CBD stones of 1, 0.9, 0.8 and 1, respectively.

**Conclusion:**

Linear EUS is safe and efficacious for the diagnosis of occult CBD stones in patients with intermediate risk for the disease.

## Background

Common bile duct (CBD) stone is a common clinical problem that can cause serious complications, such as acute cholangitis and pancreatitis [1]. Between 3 to 33% of patients with symptomatic gallstones have associated CBD stones [2].

Neither clinical/biochemical data, transabdominal ultrasound, and computed tomography (CT) can accurately predict the presence of CBD stones. The sensitivity and specificity of CT in diagnosing CBD stones are 77% and 72%. The diagnostic rate of CT is significantly lower in patients with stone size < 5 mm than in patients with stone size ≧ of 5 mm (57% vs. 81%) [3].

Endoscopic retrograde cholangiopancreatography (ERCP) remains the gold standard for both diagnosis and treatment of CBD stones; however, the procedure is associated with an overall complication rate of 5–10% and mortality rate of 0.02–0.5% [4-7]. It has been shown that the rate of post-ERCP pancreatitis may be as high as 15%, which includes 1% of patients graded as severe in degree [8]. This is true in high risk patients such as suspected sphincter of Oddi dysfunction, but not bile duct stones. Less than 67% of patients with an intermediate risk for CBD stones (occult CBD stones) require therapeutic intervention [2,9,10]. Thus, over 30% of patients with occult CBD stones do not need an ERCP exam. An accurate, safe, and efficacious method is needed to diagnose CBD stones in a definitive manner.

Magnetic resonance cholangiopancreatography (MRCP) has emerged as a non-invasive method to evaluate the biliary system [11]. It is beneficial in that it requires no sedation, involves no radiation exposure, and is free of complication. However, the equipment is rather expensive and not commonly available in every hospital. Moreover, the diagnostic rate is dramatically decreased for smaller CBD stones (≦5 mm, sensitivity: 67%) [12]. Radial echoendoscopy (EUS), on the other hand, is a minimally invasive procedure that has a low procedural risk similar to ordinary gastroscopy. It is an excellent method for examining the CBD and has been proven to have diagnostic accuracy comparable to ERCP. In addition, the CBD stone detection rates do not vary with stone size using radial EUS [13]. The images of biliary-pancreatic system and the related pathology derived from radial EUS are similar to CT scans and are convenient for guiding therapy.

Linear echoendoscopy is a newer form of EUS. As such, there is scant published information concerning its diagnostic efficacy for CBD stones. The aim of this study was to investigate the safety and diagnostic accuracy of linear EUS in detecting occult CBD stones.

## Methods

This is a prospective observational study. 30 patients (arbitrarily chosen) were recruited to determine the sensitivity, specificity, positive and negative predicted values of linear EUS for the diagnosis of occult bile duct stone, as well as, possible related adverse events.

### Patients

This prospective study was approved by the Institutional Review Board of Kaohsiung Veterans General Hospital, and written informed consent was obtained from all the participating patients beforehand.

Between February 2009 and December 2011, 30 patients with intermediate risk (<67%) for CBD stones [2] were enrolled in our study. Inclusion criteria involved a manifestation of the following set of symptoms/signs at presentation or within six months prior to admission [14] biliary colic, unexplained derangement of liver function tests (such as total bilirubin, alkaline-phosphatase, GOT (glutamic oxaloacetic transaminase)/GPT (glutamic pyruvic transaminase), and γ-GT (Gamma-glutamyl transferase)), enlarged CBD ≧ 8 mm with an intact gallbladder (GB) (≧ 10 mm in patients who had received cholecystectomy) under conventional ultrasound (US) without an identifiable cause, or any combination of the above symptoms/signs such as biliary pancreatitis.

Exclusion criteria included patients with acute cholangitis, history of gastrectomy, sphincterotomy, or sphincteroplasty, possible drug- or alcohol-related liver function impairment, history of CBD stones that had already been found by means of conventional ultrasound/CT scan, tumor of the bile duct that had already been identified, impaired consciousness, and severe cardiovascular or psychiatric diseases.

### Diagnostic and therapeutic procedures

Local anesthesia of the pharynx was performed using 10% xylocaine, and an intramuscular injection of 40 mg hyoscine-*N*-butylbromide and 25–50 mg meperidine were administered as premedication. EUS was performed using a linear-array echoendoscope (GF-C2000, Olympus Optical, Tokyo, Japan) at 7.5 MHz frequency and ERCP was performed with a side-view endoscope (JF-240; Olympus Optical Corporation, Tokyo, Japan) by the same experienced operator (Chan HH). A CBD stone was diagnosed by EUS if a persistent hyperechoic lesion was noted, with or without an acoustic shadow. Once the stone was disclosed by EUS imagery, ERCP with stone extraction was subsequently performed in the same section. This procedure was in compliance with the recently published guidelines by the American Society for Gastrointestinal Endoscopy emphasizing that CBD stones should be removed if detected unless significant mitigating clinical circumstances are present [15].

After selective cannulation of the CBD using a catheter, cholangiography was performed to confirm the diagnosis of a CBD stone. A 0.035-inch guide-wire (Boston Scientific, Corp, MA, USA) was then inserted into the bile duct through the catheter. A dilating balloon (CRE balloon, 5.5 cm in length, 0.8-1.2 cm in diameter; Boston Scientific, Corp, Ireland) was passed via the pre-positioned guide-wire into the bile duct. Using fluoroscopic and endoscopic guidance, the balloon was inflated with sterile saline solution up to the optimal size and duration (usually 3–5 min) according to the stone size and each patient’s tolerance. In order to minimize the risk of perforation, the size of the balloon must not exceed the size of the CBD. After removal of the balloon and guide-wire, the stones were removed using a Dormia basket or balloon-tipped catheter. Each patient was observed in the hospital for at least 24 hours after endoscopic treatment. Procedure-related adverse events were recorded according to the definitions and grading systems of the recent workshop held by the American Society of Gastrointestinal Endoscopy [16]. Clinical evaluation of each patient’s symptoms and serum amylase was performed the following day.

**Figure 1:**
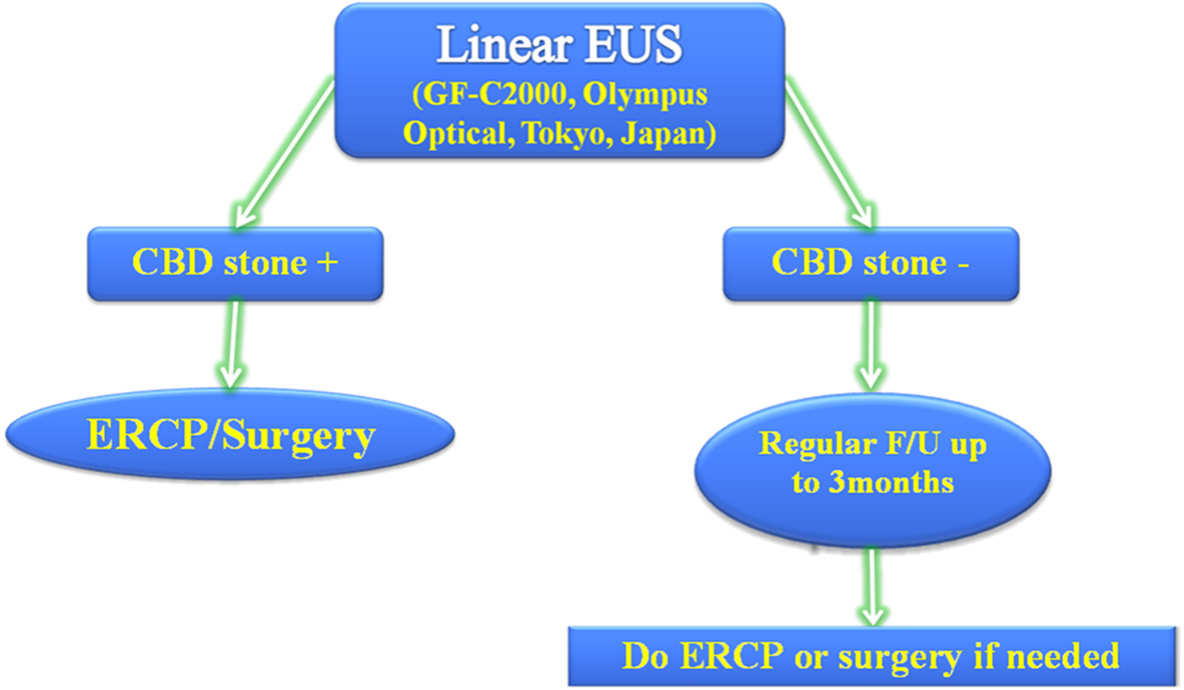
The flow-chart outlines how patients with CBD stones found by linear EUS are sent for treatment (ERCP/surgery), while patients with negative findings are followed up for up to three months.

The patients with positive CBD stones found by EUS who subsequently failed ERCP procedures were referred for surgical intervention. If no stone was found by EUS, ERCP would not be performed and patients were followed-up in the outpatient clinic or via telephone for detection of any possible biliary symptoms for up to three months. In the case of recurrence of biliary symptoms that necessitated further treatment during the follow-up period, patients were admitted for ERCP on an in-patient basis or referred for surgical treatment. Figure 1 shows a flow-chart summarizing our study plan.

Patients with stones correctly identified by EUS who were subsequently proved by ERCP or surgery were considered true positive. Patients who had no stones, but were incorrectly diagnosed by EUS and subsequently disclosed by ERCP (with bile analysis) or surgery were considered false positive. Patients who had no stones, but were correctly diagnosed by EUS and found to have no biliary symptoms during the follow-up period, were considered true negative. Patients who had stones in their bile ducts, but were missed by EUS and finally found to be symptomatic during the period of follow-up, were considered false negative.

## Results

Between February 20, 2009 and December 31, 2010, 30 patients were enrolled in the study. Characteristics of patients and reasons for their enrollment are shown in Table 1. Two-thirds of the patients were male (mean age: 60.17 ± 15.08 years). Most patients (26 out of 30) had an intact gallbladder, and among them, 15 patients had GB stones. Moreover, seven patients possessed a juxtapapillary diverticulum.

**Table 1:** Patient characteristics

**Sex(M/F)**	**20/10**
Age (mean ± SD)	60.17 ± 15.08 y/o
Intact GB (GB stone)	26 (15)
Juxtapapillary diverticulum(+/-)	7/23
**Reasons for inclusion**	**Number of patients**
Acute pancreatitis	15
Abdominal pain	1
Abdominal liver function	1
Dilated bile duct	2
Abdominal pain + Abnormal liver function	5
Abdominal pain + dilated bile duct	2
Abdominal pain + Abnormal liver function + dilated bile duct	4

The major reason for enrollment was acute pancreatitis (15 patients). In addition to pancreatitis, these 15 patients presented with at least one or more of the following signs of CBD stones, which include: jaundice (eight patients), dilated CBD (five patients), GB stones (nine patients) and elevated Alkaline-phosphatase and Gamma-glutamyltransferase (10 patients). No direct evidence of CBD stones was found by means of transabdominal ultrasound (30 patients) and CT scan (24 patients, others did not receive CT scan exam). The mean predicted risk for CBD stone was 47% (28–61) [9].

**Figure 2:**
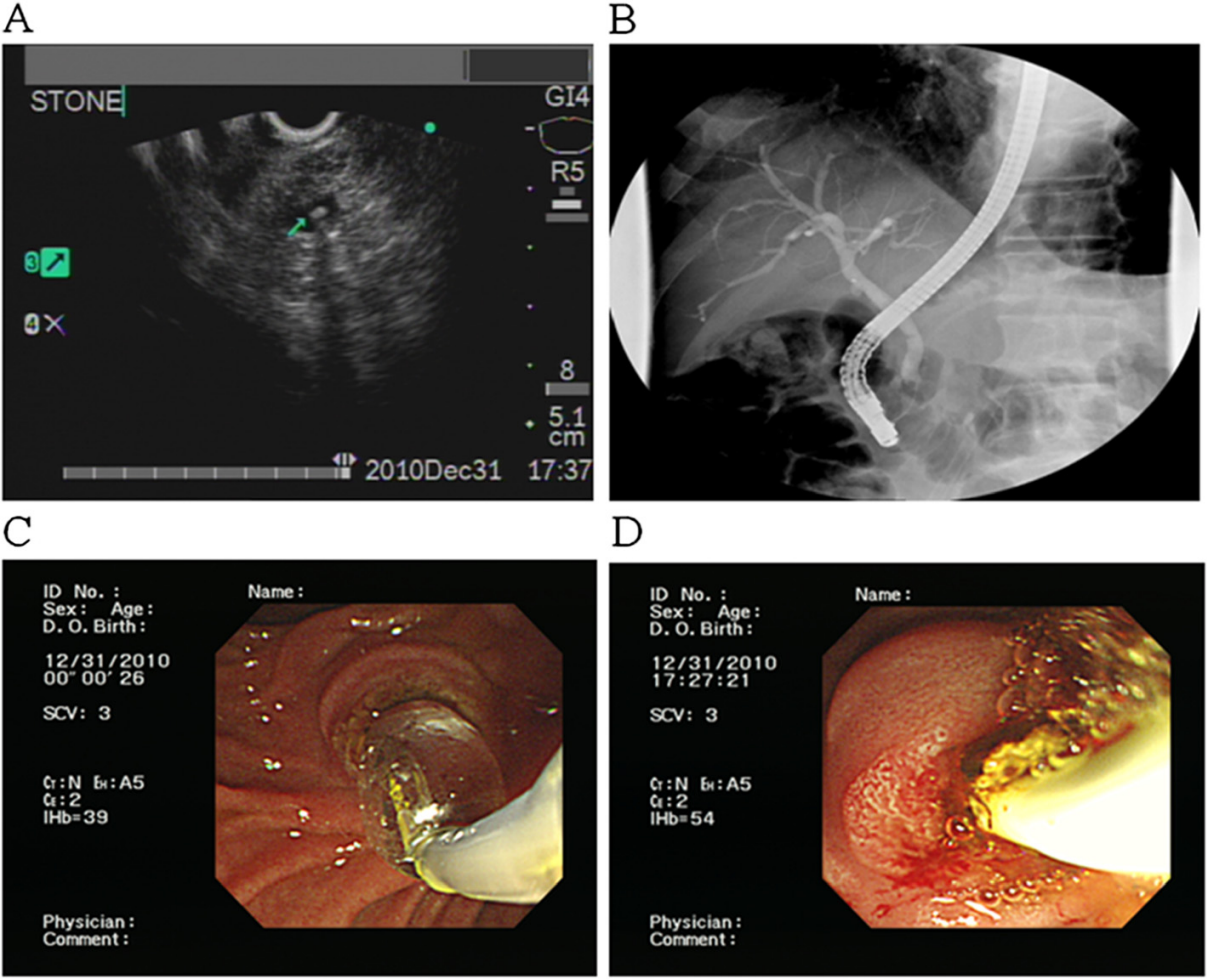
**(A) A tiny CBD stone is revealed using linear EUS. **(**B**) However, no definite filling defect is seen in the ERCP picture. (**C**) and (**D**) A tiny yellowish CBD stone is extracted after balloon dilation is applied.

**Figure 3:**
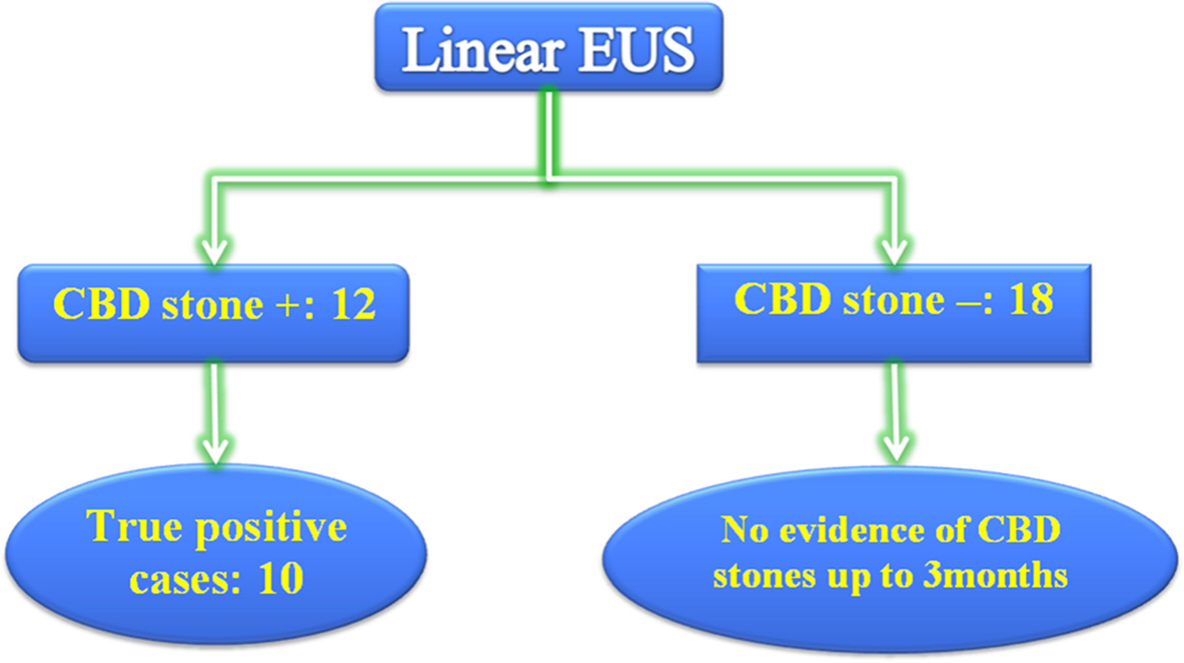
**12 patients were positive for CBD stones found by linear EUS. **10 were true positive. 18 patients were negative for CBD stones and no false negative noted during the 3 months of follow-up.

Of the 12 patients who were positive for CBD stones by EUS (Figure 2), eight were treated successfully using ERCP in the same section. One patient, who had failed in the same section of EUS, was treated successfully by ERCP 32 days later following the refusal of surgery by patient. Another one failed ERCP and was later treated successfully by surgical extraction. There were two false-positive cases. In one, no gross stone was extracted during ERCP procedure, and no stone crystal was found in the aspirated bile under the microscopic exam. Therapeutic ERCP failed for the other case and the absence of a stone was proved by surgery. All detected stones were ≦ 5mm in size except one (which measured 0.85 mm in diameter). All patients tolerated both endoscopic procedures well and no EUS or ERCP related adverse events were noted. For those 18 patients who had no evidence of stones by EUS, no false-negative case was noted during the three- month follow-up period (Figure 3).

In the current study, sensitivity, specificity, positive and negative predicted values of linear EUS for the detection of occult CBD stones were 1, 0.9, 0.8 and 1, respectively.

## Discussion

Systematic review has shown that there was no significant difference between EUS and MRCP for the detection of choledocholithiasis [11]. The choice of the equipment depends on availability, physician’s experience, and cost considerations.

MRCP is, theoretically, more objective in the diagnosis of biliary diseases than EUS or ERCP. However, patients with claustrophobic tendencies may obviate its use. In addition, MRCP requires sophisticated maintenance, which is more costly to operate. It is available only in medical centres where a group of experienced radiologists team up to interpret the MRCP images. MRCP is especially useful for potentially occult CBD stones that fail to be discovered by conventional ultrasound or CT scans, as in our cases. However, the different properties of MRCP compared to EUS/ERCP require a different type of patient preparation, rendering it inconvenient to perform subsequent therapeutic ERCP immediately after MRCP, in the event that CBD stones are identified. On the other hand, linear EUS, compared to MRCP, is more portable and less costly to operate. In addition, although the learning curve for linear EUS is steep, it is economically feasible to train a small group of endoscopy fellows within the same hospital to perform the procedure. Quality images can be obtained from linear EUS and they can be further improved by adjusting either the contrast or brightness in real time, as well as by manipulating the relative distance, location and direction of the tip of the endoscope to the target lesion. It is also easy to interpret linear EUS images due to the proximity of the EUS probe to the CBD, which excludes intestinal gas interference. Although, linear EUS is more invasive than MRCP, the procedure-associated risk of performing linear EUS is similar to ordinary upper gastrointestinal endoscopy and lower compared to ERCP.

A previous report [14] has shown that linear EUS is a reliable method for the evaluation of patients with high risk for CBD stones. Furthermore, it has been previously reported [17] that a considerable portion of patients with intermediate risk of CBD stones (as in our current study consisting of a group of patients with no direct evidence of CBD stone found by transabdominal ultrasound/CT scan) have no evidence of stones by linear EUS, thus, avoiding unnecessary invasive evaluation of the bile duct with ERCP.

National health policy has a major impact on our study. In Taiwan; all citizens are included in National Health Insurance. Each citizen and their family receive fairly effective medical care in exchange for less than 10% of their salary. However, this poses a large financial burden on the government. It is, therefore, not cost-effective to administer conscious sedation to every patient receiving an endoscopic exam. This financial consideration has a psychological impact on patients experiencing the necessity to change to the side-view duodenoscope when they needed a therapeutic ERCP immediately after CBD stones were found by EUS.

In addition, there is no “gold standard” for the detection of CBD stones in the study. Since ERCP has been performed only in patients with positive CBD stone by EUS and follow-up of patients has, in part, been conducted just by telephone, the rate of false negative findings may be underestimated. Another shortcoming of the study is that both EUS and ERCP were performed by the same investigator, and no blinding has been performed.

## Conclusions

This study suggests that linear EUS can accurately detect CBD stones in patients with intermediate risk for the disease, when conventional imaging techniques have failed. This procedure can, therefore, minimize the number of unnecessary invasive ERCP procedures for this subset of patients. There were no procedural-related adverse events caused by the use of linear EUS for the diagnosis of CBD stones. However, one drawback to our study involved the need to change to the side-view duodenoscope when patients required ERCP treatment. Hopefully, future technological advancement will provide an endoscope that enables performance of both EUS and ERCP.

## Competing interests

The authors declare that they have no conflicts of interests.

## Authors’ contributions

HHC, MSS, PIH, KHL and WRB designed the study and analyzed the data. WRB taught HHC how to do EUS when HHC was a research fellow in Massachusetts General Hospital during the year of 2002. HHC, KHL, and EMW were responsible for writing the manuscript and revising it critically for important intellectual content. HHC was responsible for the EUS and ERCP procedures. EMW, TJT and KMW assisted the endoscopic procedures. HHC, WLT, WCC and HMW were responsible for patient care. HLL was responsible for the ultrasound and CT scan interpretation. All authors have read and approved the final manuscript.

## Pre-publication history

The pre-publication history for this paper can be accessed here:

http://www.biomedcentral.com/1471-230X/13/44/prepub
